# Effects of resistance exercise in elderly cancer patients

**DOI:** 10.4314/ahs.v23i2.34

**Published:** 2023-06

**Authors:** Jing Sun, Li Ge, Chunjuan Cao, Wen Yao, Xiaoling Wang

**Affiliations:** 1 Department of Oncology, Xinhua Hospital Affiliated to Shanghai Jiao Tong University School of Medicine; 2 Emergency Medicine Department, Xinhua Hospital affiliated to Shanghai Jiao Tong University School of Medicine; 3 Nursing Department of Xinhua Hospital Affiliated to Shanghai Jiao Tong University School of Medicine; 4 Department of Endocrinology, Xinhua Hospital affiliated to Shanghai Jiaotong University School of Medicine

**Keywords:** Cancer patients, elderly, resistance exercise

## Abstract

**Objective:**

To investigate the effects of resistance exercise (RE) in elderly cancer patients (ECPs).

**Methods:**

Convenience sampling was used to select 240 ECPs, who received radiotherapy and chemotherapy at the Department of Oncology of a Level-A tertiary hospital in Shanghai from September 2019 to August 2020, and they were randomized to control (CON) and experimental (EXP). After a 12-week intervention, the Functional Assessment of Chronic Illness Therapy-Fatigue (FACIT-F), the 36-Item Short Form Health Survey (SF-36), the Connor-Davidson resilience scale (CD-RISC) and the Functional Independence Measure (FIMSM) were used to evaluate the effects of RE in two groups.

**Results:**

After 12-week intervention, the total FIMSM score and the scores of self-care, transfers, locomotion, communication, and social cognition of EXP was higher than before, and compared with CON (P<0.05). However, the intervention did not effectively improve the sphincter control (P>0.05). The SF-36 and CD-RISC scores of EXP were higher than those of CON, and FACIT-F score was lower than that of CON with statistically significant differences (P<0.05).

**Conclusion:**

RE effectively relieved the cancer-related fatigue (CRF) in ECPs, improved their mental state, quality of life and mobility.

## Introduction

According to a WHO report, there are 8.8 million deaths in the world every year, among which 60.06% of the new cases are over 60 years old, and 61.11% of the new cancer patients in China are over 60 years old 1. With the development of cancer treatment, the number of elderly cancer survivors increases year by year continuously 2. Physical and psychological adverse reactions accompany many problems 3. Among them, the most common are cancer-related fatigue (CRF) 4. CRF is the most common adverse reaction of cancer patients and survivors, and may lead to heavy economic burden on family and society 5.

Resistance exercise (RE) has been widely used in cancer patients and proves to have positive effects. Studies have shown that the application of RE in cancer survivors can effectively improve their quality of life and prolong their lives. RE can better the mental state and quality of life of cancer patients, improve physical comfort and function, reduce their CRF and change biochemical indicators (BIs) related to cancer progression 6,7 Present study aims to explore the safe application of resistance exercise in elderly patients, and to effectively reduce cancer-related fatigue, improve the clinical outcome, quality of life, physical function, and psychological status of patients by carrying out the intervention.

## Material and Methods

### General information

Two hundred and forty cancer patients at the Department of Oncology of a Level-A tertiary hospital in Shanghai were selected by convenience sampling as the research subjects from September 2019 to August 2020, and randomized into experimental (EXP) and control (CON). In CON, there were 58 males (48.3%) and 62 females (51.7%), aged 50~87 (67.10±5.76) years old, with the disease duration ranging from 0.5 to 20 years. Seventeen patients (14.2%, 17/120) had disease duration of over 5 years, and 31.7% (38/100) had recurrence. In EXP, there were 57 males (47.5%) and 63 females (52.5%), aged 50~87 (63.68±7.01) years old, with the disease duration ranging from 0.5 to 18 years, and 20 patients (16.7%, 20/120) had the disease duration of over 5 years, and 37.5% (45/100) patients had recurrence. The exercise mode of patients in both groups was mainly walking in public places. There were no significant differences (P>0.05) in general information, such as gender, age, disease duration, exercise time and mode between the two groups, that indicates comparability.

### Inclusion and exclusion criteria

**Inclusion criteria:** (1) cancer patients equal or greater than 60 years old; (2) with relatively good anti-cancer treatment effects, in stable condition and with over 1 year expected survival; (3) fit for RE; (4) with clear mind, and can fully express their own opinions; (5) volunteering to participate in this study.

**Exclusion criteria:** (1) patients who are undergoing psychological intervention or other sports intervention; (2) with skeletal muscle diseases; (3) with severe cardiovascular diseases; (4) with acute or moderate-to-severe non-diabetic nephropathy; (5) undergoing lymphedema examination and treatment, cancer survivors with lymphedema; (6) with bone metastases, and patients with possible pathological fractures diagnosed by doctors.

### Develop exercise rehabilitation program

The RE program for ECPs based on the literature review, including the application effect of RE on ECPs and the exercise prescription of ECPs was preliminarily developed. Expert groups were invited to revise exercise program, define exercise evaluation index, and add health education and promotion and other related content. The group included 7 experts in clinical medicine (Department of oncology, rehabilitation medicine, neurology and neurosurgery), nursing management, clinical nursing (Department of oncology and rehabilitation medicine) and other fields (Table 1). Expert judgment basis (Ca), expert familiarity (Cs), and expert authority (Cr) were 0.97, 0.91, and 0.94 respectively. The experts' opinions were summarized and the following questions were put forward: First, the subjects under intervention were unclear and not focused, and the inclusion and exclusion criteria needed to be further improved; Second, there were some deficiencies in the content of the intervention, namely exercise mode, including the exercise taken, the amount, time, frequency and goal, which needed to be improved; Third, patients' safety and exercise compliance during intervention needed to be ensured.

**Table 1:** General Information of experts

SN	Gender	Age	Education	Title	Duration of working life	Specialty
1	Female	39	MD	associate professor of treatment	16	associate professor of rehabilitation medicine
2	Female	52	BD	associate professor of nursing	35	nursing management
3	Male	45	Ph. D	associate professor of treatment	15	associate professor of neurosurgery
4	Female	49	BD	associate professor of nursing	30	nursing management
5	Female	49	MD	associate professor of treatment	26	associate professor of neurology
6	Male	41	Ph. D	associate professor of treatment	19	associate professor of oncology
7	Female	49	BD	associate professor of treatment	26	associate professor of oncology

### Program Implementation

**Control (CON) Routine nursing:** routine diet and drug guidance during radiotherapy and chemotherapy.

### Experimental

#### Theory teaching

The CRF knowledge, dietary guidance during radiotherapy and chemotherapy, and the importance, benefits and methods of RE with pictures, words and videos in the oncology ward have explained. Since the patients were old, they were accompanied by their families when coming for hospitalization. Therefore, encouragement the family members to join RE to accompany, encourage and supervise the patients, and also have given patients guidance when discharged.

#### Exercise prescription

The patients in CON performed RE on the basis of routine chemotherapy nursing, which mainly included three steps: pre-exercise, exercise and exercise recording 8.

(1) In pre-exercise, we explained the specific requirements of RE to patients and let them watch exercise videos; then evaluated their resistance strength, and set individual strength and elastic band according to the results. The set strength is 30 to 50% of the maximum load.

(2) In exercise step, elastic band, which was selected according to the baseline of patients after admission, was mainly used. The exercise lasted 40 min, including warmup and relaxation each for 5 min before and after, and RE for 30 min. They were required to do 2~4 sets each time, with 10-15 repetitions (reps) gradually reduced to 8~12 times in each set and with 2~3 min intervals; the exercise gradually increased from 10~15RM (repetition maximum) to 8~12RM. They did it for 12 weeks with five times a week (Wednesday and Sunday off).

(3) In exercise recording, instruction has been given to the patients to keep RE diaries, including RE mode, time, number of sets and reps, intensity and their feelings during exercise.

**Follow-up call:** We made weekly calls to the patients, answered their questions and urged them to exercise and record the diary on time, and gave them support and encouragement.

**Outpatient follow-up:** Each time the patients came to the hospital for a return visit, the researchers collected and examined RE diaries and talked with the them about how they felt during exercise, encouraging them to exchange their exercise experiences with each other.

#### Evaluation index

**CRF:** In this study, FACT-F was adopted to assess CRF of ECPs. It had 41 items in total, and a 4-point rating scale was used, with 0 representing “not at all” and 4 representing “very much”, with a total score of 0-164 10. Yellen et al. 9 formed FACT-F by adding 13 items on the basis of FACT-G 12. The 13 additional items can be used as an independent fatigue scale, which has been validated in patients with different cancers and anemia with good psychometric attributes and internal consistency of 0.93~0.95.

**Quality of Life:** Quality of life was assessed using the internationally recognized SF-36. The scale includes several items and questions. The score is calculated according to the selected answers. The higher the score is, the better the health is 11.

**Psychological resilience:** Connor and Davidson developed CD-RISC, which has 25 items in total, including 5 factors: personal competence, tolerance of negative affect, acceptance of change, sense of internal control, and spirituality. The Chinese version of CD-RISC was translated and revised by Xiao Nan, it retained 25 items of the original scale 12, which were adjusted into three factors of tenacity, strength and optimism. The Cronbach's α of the Chinese version was 0.91 and it is widely used to monitor the psychological resilience of the patients 13.

**Physical Function:** We used FIMSM to test the physical function. The higher the score, the more independent the patient is in performing the task associated with that item. It includes measures of independence for self-care, sphincter control, transfers, locomotion, communication and social cognitions 14.

**Data collection:** The researchers received unified training, and the patients filled in the form of general information, questionnaires at the time of admission. Meanwhile, the researchers assessed the patients' exercise status and developed personalized exercise programs. After the 12-week intervention, those indexes were collected and assessed again.

### Statistical Methods

Valid questionnaires were entered into the database by two experts. After their review, SPSS22.0 was used for statistical analysis. T test was used. P<0.05 was statistically significant.

## Results

The comparison of the scores of questionnaires between two groups before and after intervention has been analysed and reported (Table 2).

**Table 2:** Comparison of the scores of questionnaires before and after intervention in two groups (N=120, point, X̅±S)

questionnaires	Group	CON		EXP	

		Before	After	Before	After
scores of physical functions	Total	109.81±9.97	112.03±10.3 4	111.03±10.34	117.70±10.38^a, b^
	Self-care	36.15±5.93	37.13±5.17^a^	37.21±4.79	38.74±3.09^a, b^
	Sphincter control	11.73±1.83	12.07±1.61	11.97±1.69	11.94±2.31
	Transfers	18.71±2.43	18.92±2.63	18.74±2.62	19.41±2.30^a, b^
	Locomotion	11.93±2.23	11.70±1.91	12.05±2.06	13.55±1.83^a, b^
	Communication	11.97±1.84	12.05±1.65	12.18±1.86	12.52±1.82^a, b^
	Social cognition	15.91±2.91	18.28±2.48^a^	18.70±1.95	19.13±1.83^a, b^
psychological resilience	Total	78.75±9.72	83.61±8.10^a^	77.70±11.20	88.59±6.16^a, b^
	Optimism	12.53±2.10	13.11±2.34^a^	12.51±2.28	14.53±2.31^a, b^
	Tenacity	40.61±5.63	43.01±6.12^a^	40.10±1.69	45.27±5.01^a, b^
	Strength	25.62±3.36	27.49±3.54^a^	25.38±3.84	28.78±2.88^a, b^
FACIT-F	total score	109.36±25.89	86.54±15.14^a^	107.09±28.31	81.13±14.81^a^
SF-36	total score	88.23±5.64	94.06±9.69^a^	89.57±5.74	97.92±6.98^a^

a compared with the score before intervention, *P*<0.05

b compared with the score of CON, *P*<0.05

## Discussions

High death rate and high treatment cost bring serious psychological and physical burden to cancer patients and their families. Surgery combined with radiotherapy and chemotherapy is the main treatment for cancer patients, but it will further increase their burden 15. CRF is one of the most common adverse reactions in cancer patients, especially in elderly ones. The latest CRF management protocol National Comprehensive Cancer Network (NCCN) of the US clearly points out that CRF should be managed from four aspects: screening, preliminary assessment, intervention and reassessment 4, and exercise therapy is one of the most important non-drug treatments for cancer patients, which can effectively relieve their fatigue and improve their psychological state.

The systematically evaluated the effect of RE in ECPs and designed the RE prescription combined with relevant guidelines and expert advisory, and the patients in EXP were required to perform a 12-week RE on the basis of routine nursing and treatment plan. The results showed that FACIT-F score decreased after intervention, which meant RE can effectively relieve the fatigue of patients and improve their mental health. Further analysis showed that RE significantly improved physical and mental fatigue and increased mobility (P<0.05), but did not alleviate motivation fatigue (P>0.05). RE can reduce the activation of microglia and increase the expression of neurotrophic factors such as brain-derived neurotrophic factor and adiponectin, which can protect the brain and effectively relieve mental fatigue of patients, whereas, moderate regular exercise can promote the secretion of endorphins in the brain. With the effect of hormones, it can relieve tension, inner pressure and unpleasant feelings, and help maintain a healthy mental state 16.

RE is an active body movement that the muscle overcomes the external resistance repeatedly and regularly in order to recover and improve the muscle strength, and carry out the recovery training of muscle atrophy. Due to disease and the influence of radiotherapy and chemotherapy, patients suffer from impaired immune nervous system, disorder of self-image and other physical and mental problems, resulting in limited quality of life index and difficulty in participating in normal social life 17. According to the results of the physical function evaluation, there were statistically significant differences in self-care, transfers, locomotion, communication and social cognition in EXP compared with those in CON (P<0.05), and the differences before and after intervention were statistically significant (P<0.05). Therefore, RE can effectively better patients' muscle function, locomotion (13.55±1.83, P<0.05) and transfers (19.41±2.30, P<0.05), improve body strength and physical quality (P<0.05), which is consistent with the research results of Santos et al. 18. At the same time, it can effectively shift their attention and boost their confidence in exercise. Moreover, it provides the patients with more topics to talk with their family or surrounding, promotes the communication with others, and thus improves their social cognition and the quality of life.

Psychological resilience focuses on people's physical and mental positivity, and then stimulates people's potential strength to overcome adversity. In the course of treatment, cancer patients suffer from depression, anxiety and mood disorders, and their psychological status and quality of life are seriously affected. Healthy living helps patients overcome the disease and bring hope and confidence. Studies on psychological resilience show that good social environment and family interpersonal relationship are conducive to relieve their psychological burden and improve their mental resilience 19. Exercise therapy can help relieve perennial negative emotions such as fatigue and anxiety, help patients lead a healthy lifestyle, reduce hospital stay and medical costs, and improve the quality of life 20, 21. The results of our study were similar to those of others. The total score (88.59±6.16, P<0.05) of psychological resilience and the scores of all measures after intervention in EXP increased compared with CON and before intervention, and the results were statistically significant (P<0.05). Therefore, RE can effectively better the quality of life and the mental resilience of the patients, thus improving their psychological state.

## Future Prospects

At present, although there are many studies on the application of RE in cancer patients, no consensus has been reached on exercise prescription such as mode, intensity, duration and frequency 22. The present investigation concluded the following problems during the intervention and proposed the following suggestions:

1. If there were no continuous and regular follow-up intervention, patients' exercise compliance will gradually decrease with the time of discharge and the interruption of intervention. Therefore, our research center should focus on how to make patients exercise regularly and improve their exercise compliance in the future.
2. Regular RE can effectively improve the clinical outcome, but patients' awareness of exercise mode, frequency and intensity are not strong, and the specific rules are unknown to them. Therefore, it is of great significance to strengthen the education of exercise and disease knowledge, and then promote the relevant knowledge of patients.
3. Most patients had misunderstood that exercise increased fatigue and worsened health conditions with many security issues and so on. Therefore, it needs to actively evaluate the overall situation of patients, develop personalized RE program for ECPs, strengthen the awareness of exercise rehabilitation of the patients and their families, so that they are willing to start and continue to exercise in a safe environment, and finally get a better quality of life.

## Conclusion

In conclusion, the current CRF and psychological resilience of ECPs are worrying, and exercise therapy can effectively reduce CRF and improve the physical function of patients, and then their quality of life and psychological state were improved.

## Declarations

### Availability of data and materials

The datasets analysed in this case report are available from the corresponding author on request.
